# Decombinator V4: an improved AIRR-C compliant-software package for T-cell receptor sequence annotation?

**DOI:** 10.1093/bioinformatics/btaa758

**Published:** 2020-08-27

**Authors:** Thomas Peacock, James M Heather, Tahel Ronel, Benny Chain

**Affiliations:** Division of Infection and Immunity, UCL, WC1E 6BT, London, UK; CoMPLEX, Department of Computer Science, UCL, WC1E 7JG, London, UK; Massachusetts General Hospital Cancer Center and Harvard Medical School, Boston, MA 02115, USA; Division of Infection and Immunity, UCL, WC1E 6BT, London, UK; Cancer Institute, UCL, WC1E 6DD, London, UK; Division of Infection and Immunity, UCL, WC1E 6BT, London, UK; CoMPLEX, Department of Computer Science, UCL, WC1E 7JG, London, UK

## Abstract

**Motivation:**

Analysis of the T-cell receptor repertoire is rapidly entering the general toolbox used by researchers interested in cellular immunity. The annotation of T-cell receptors (TCRs) from raw sequence data poses specific challenges, which arise from the fact that TCRs are not germline encoded, and because of the stochastic nature of the generating process.

**Results:**

In this study, we report the release of Decombinator V4, a tool for the accurate and fast annotation of large sets of TCR sequences. Decombinator was one of the early Python software packages released to analyse the rapidly increasing flow of T-cell receptor repertoire sequence data. The Decombinator package now provides Python 3 compatibility, incorporates improved sequencing error and PCR bias correction algorithms, and provides output which conforms to the international standards proposed by the Adaptive Immune Receptor Repertoire Community.

**Availability and implementation:**

The entire Decombinator suite is freely available at: https://github.com/innate2adaptive/Decombinator.

**Supplementary information:**

Supplementary data are available at *Bioinformatics* online.

## 1 Introduction

The analysis of T**-**cell and B**-**cell antigen**-**receptor sequences is challenging because the final somatic DNA sequences from which the receptors are transcribed and then translated are produced after a series of cell intrinsic recombination events which irreversibly change the somatic genome. Decombinator was one of the early Python software packages released to process the rapidly increasing flow of new T**-**cell receptor repertoire sequence data, and to infer the precise set of recombination events which give rise to each sequence read (Thomas *et al.*, 2013). The strategy underlying Decombinator is to use a finite state machine to classify individual TCR sequences using a set of molecular tags uniquely matching individual V and J regions of the T**-**cell locus. It is based on the Aho-Corasick algorithm (Aho *et al.*, 1975) which remains the fastest way to execute exact multiple keyword searches. The algorithm uses a pre-constructed keyword trie to match a set of queries to targets. The speed scales linearly with the number of keywords and the length of the target string, thus substantially outperforming commonly used local alignment algorithms, especially when the number of keywords becomes large. Decombinator has been freely available on GitHub since its original publication, and has been frequently extended and modified. The name Decombinator originally referred specifically to the Python module which inferred the underlying recombination events giving rise to each read. However, the same name has now been extended to refer to a whole package of modules, of which the original Decombinator is one. The package now incorporates additional modules providing multiple sample demultiplexing based on sample-specific dual barcoding (Demultiplexor), unique molecular identifier (UMI)-based functions to correct for sequencing error and PCR bias (Collapsinator), and translation modules to provide CDR and full**-**length TCR amino acid sequences (CDR3Translator). Although the package has been used in several published studies, e.g. (Gkazi *et al.*, 2018; Heather *et al.*, 2016; Joshi *et al.*, 2019; Oakes *et al.*, 2017; Wong et al., 2018), none of the changes to the original Decombinator have been formally published. In this short report, we document some major changes to the Decombinator package, which we have released on GitHub as Decombinator V4 (https://github.com/innate2adaptive/Decombinator).

Decombinator V4 has been ported to Python 3, since support for Python 2 ended in 2019. We have redesigned the error correction algorithms in Collapsinator, resulting in significantly improved robustness to PCR/sequencing error correction based on UMI clustering. This is described in more detail below. Importantly, we have completely reconfigured the output of the CDR3Translator module, so the final output is now fully compliant with the International Adaptive Immune Receptor Repertoire (AIRR) Community recommendations (https://docs.airr-community.org/en/latest/). This greatly improves the utility of Decombinator, as it ensures the output is compatible with the growing number of secondary repertoire analysis tools which are being released.

## 2. Results and discussion

Full details describing installation and use of the four Decombinator modules are available on GitHub (https://github.com/innate2adaptive/Decombinator), together with some test datasets. Instead, we focus on evaluation of the most significant development since our original publication, the UMI-based PCR bias and sequencing error correction function of the Collapsinator module, and we briefly outline the output fields produced by CDR3Translator.

We first compared the output of Decombinator on a simulated ‘ground truth’ set of TCRs generated using IGoR (Marcou *et al.*, 2018) as described in Supplementary Methods. Decombinator V4 correctly annotated 92.7±0.5% (mean±standard deviation) of the sequences. CDR3 sequences were correctly identified in 98.1±0.5% of the sequences. The majority of miss-annotations occurred in relation to very similar sub-families of V region (e.g. TRBV6-1, TRBV6-2, TRBV6-3; or TRB12-1, TRBV12-2) between which the Decombinator tags do not distinguish.

**Fig. 1.**
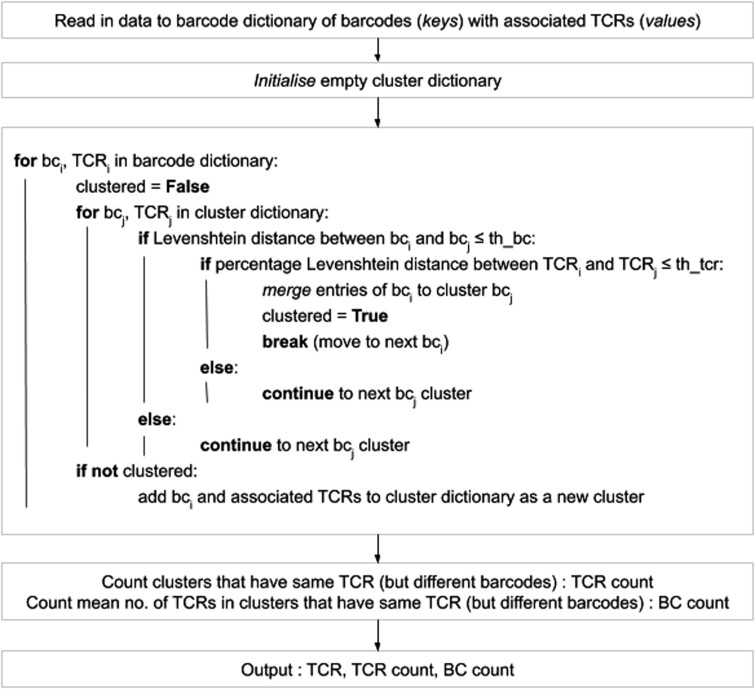
Pseudocode outlining the main functionality of the Collapsinator script. TCR data in the Decombinator format is read into the program and initially grouped by barcode. Each of these groups undergo pairwise comparison, whereby the barcode (bc_i_) and the most frequent TCR sequence (TCR_i_) of group i is compared to the barcode (bc_j_) and the most frequent TCR sequence (TCR_j_) of group j. If barcodes bc_i_ and bc_j_ are similar relative to the barcode threshold (th_bc), and sequences TCR_i_ and TCR_j_ are similar relative to the sequence threshold (th_tcr), then groups i and j are merged. The merged groups are here referred to as clusters. Similarity measures are taken as the Levenshtein distance for barcodes, and a percentage-based Levenshtein distance for TCR sequences (Levenshtein distance weighted by length of sequence). The two thresholds are user-configurable. Once every group has been clustered, the TCR identifying classifier (V gene, J gene, no. of V deletions, no. of J deletions, insert sequence) of each TCR in the biological sample is output to file, accompanied by the number of times that TCR was found in the sample (TCR count) and the mean cluster size (BC count) associated with that TCR

A pseudocode description of the Collapsinator module is shown in Figure 1, and is discussed in more detail in Supplementary Methods. We evaluated the error correction performance of Collapsinator by simulating PCR amplification and Illumina sequencing of the ‘ground truth’ TCR repertoires, and then measuring how well Collapsinator recovered the sequences and their abundances present before PCR (see Supplementary Methods). From 10,000 sequences present before PCR/sequencing, all but 2±1 (mean±standard deviation) were recovered by Collapsinator. A small number of sequences (109±15) were introduced by PCR or sequencing error and were erroneously retained by Collapsinator even after error correction. The correlation coefficient between the abundances of the original set of sequences, and the output of Collapsinator was 0.97±0.7. By comparison, Collapsinator V3 (the previous version) introduced 5855±335 new ‘error’ sequences, and the correlation coefficient was 0.86±0.006.

The CDR3Translator module has also been substantially rewritten. The output is a tab separated file, in the AIRR Community format (Vander Heiden *et al.*, 2018), in which each row represents a unique DNA sequence defined by Decombinator. Since the same amino acid sequences can be coded for by different DNA sequences, the amino acid sequences encoded by each row are not necessarily unique. Mandatory AIRR columns include V and J genes, using IMGT nomenclature (but note that the current Decombinator version does not distinguish alleles), the CDR3 sequence (or more specifically the ‘junction’ rather than CDR3), which includes the conserved bracketing C and F residues (Lefranc, 2014), and the number of times the TCR was recorded in the initial dataset. Additional columns include CDR1 and CDR2 sequences, as defined by IMGT, and the complete DNA and protein sequence excluding the leader sequences. A False/True flag identifies all the non-productive sequences identified, which are included in the output file by default. The format permits additional non-required fields, which we use to output information such as the traditional five-part Decombinator classifier, facilitating comparisons with the output of previous versions. Finally, each TCR is associated with a mean cluster size (BC count, see Fig. 1), which can be used to estimate the robustness of the data for that particular sequence.

In conclusion, we report the release of Decombinator V4 for the rapid and accurate annotation of TCR sequence data. Although designed to work optimally on data obtained by the experimental pipeline library preparation protocol we developed (Oakes *et al*., 2017; Uddin *et al.*, 2019), Decombinator is broadly applicable to a variety of TCR sequencing protocols. Furthermore, compliance with AIRR Community output standards will ensure that the data produced by Decombinator can be readily used by the growing number of TCR analysis tools now available to the Immunological community.

## Supplementary Material

btaa758_Supplementary_Data
