# Corrigendum to: Decombinator V4: an improved AIRR-C compliant software package for T-cell receptor sequence annotation

**DOI:** 10.1093/bioinformatics/btab550

**Published:** 2021-12-13

**Authors:** Thomas Peacock, James M Heather, Tahel Ronel, Benny Chain

*Bioinformatics* (2020) doi: 10.1093/bioinformatics/btaa758

“The title of this paper was originally, erroneously displayed as “Decombinator V4: an improved AIRR compliant-software package for T-cell receptor sequence annotation?”. This should have appeared as “Decombinator V4: an improved AIRR-C compliant software package for T-cell receptor sequence annotation. This has now been corrected online”.

