# Supplementary material for: Decombinator V4: an improved AIRR-C compliant-software package for T-cell receptor sequence annotation?
Source: Bioinformatics. 2020 Aug 27;37(6):876–8. doi: 10.1093/bioinformatics/btaa758 (PMC8098023; doi:10.1093/bioinformatics/btaa758)
Supplement: btaa758_Supplementary_Data [file btaa758_supplementary_data.zip › Supplementary Materials and methods.docx]

**Supplementary Materials and methods**.

**Simulated TCR repertoires for error correction.**

A general problem in TCR repertoire sequencing is that there is rarely a “ground truth” which can be used to robustly test new or modified pipelines (Heather *et al.*, 2018). In order to test the accuracy of Decombinator, and the error correction module Collapsinator, we have therefore generated artificial data sets by simulation. We start with a basic sequence set created using the IGoR software package (Marcou *et al.*, 2018). IGoR uses a Bayesian approach to learn probabilities from real repertoire data, and then uses them to generate new repertories which faithfully reflect many of the features of real TCR repertoires, such as the V and J gene distribution, and the gene-specific distributions of deletions and insertions. We gave individual generated TCR sequences different abundances, based on the observed long-tailed abundance distributions frequently observed in real world data (Oakes *et al.*, 2017b). Each replicate of each unique TCR sequence was then associated with a stochastically generated 12 base pair unique molecular identifier as occurs experimentally in our published library preparation protocol for TCR sequencing (Uddin *et al.*, 2019). This set of 10,000 artificially generated TCR-UMI combinations acted as our “ground truth”. Since the output of IGoR defines each TCR annotation, in terms of V, J, and CDR3 sequence, we could compare the output of Decombinator directly to the ground truth to assess the accuracy of Decombinator annotations.

In order to test the performance of the error correction in Collapsinator, we then simulated PCR amplification of our ground truth set, using a polymerase error rate of 5 * 10^-6^, somewhat higher than estimates of the *Pfu*-derived proof-reading polymerase used in our experimental pipeline. The R script for the PCR simulation is shown in Supplemental File 1. Starting with 10,000 sequences, we simulated up to 12 cycles of PCR generating a dataset of 2-3 million sequences. Finally, we used the ART Illumina simulator (Huang *et al.*, 2012) to simulate sequencing on NextSeq, which is the platform we routinely use for our in house TCR repertoire sequencing. The simulator introduces sequencing errors, and base pair quality profiles learnt from our own real world data to produce a simulated FASTQ file. The parameters used for the simulation are

art_illumina -1 NS200.txt -amp -k 1 -na -i *input_filename.fasta*  -l 200 -f 1 -o *output_filename*

The final file contains a set of approximately 2.8 million TCR sequences, reflecting typical samples that we obtain experimentally. We repeated this procedure ten times, and the mean and standard error of the performance are provided.

**Setting the UMI distance threshold.**

The incorporation of UMI in the first step of the library preparation allows both sequence error correction and correction for PCR bias. Collapsinator clusters TCRs both by UMI and by TCR sequence (Fig 1). Since the theoretical UMI count is very large (4^12^) the probability of two RNAs receiving the same UMI are small, and all TCRs carrying the same UMI can be assumed to be derived from the same PCR template molecule. Furthermore, UMI that are similar and are associated to a similar TCR are likely to be amplified from the same template molecule, and to differ only due to PCR or sequencing error. The probability of observing two barcodes which are within a Hamming distance of *d* is given by equation 1:

Equation 1 $p_{1}=Pr\left( X\leq d \right)=\sum_{i=0}^{d} \left( \begin{aligned} 12 \\ i \end{aligned} \right)\left( 0.75 \right)^{i}\left( 0.25 \right)^{12-i}$

The cumulative distribution of a binomially distributed random variable *X* representing the distance between any two barcodes, with number of trials=12 and probability of success=0.75 (success being a mismatch at each position), since each barcode is 12 base pairs long, and there are four possible nucleotides at each position. We have set the default minimum distance to d = 2, which corresponds to a probability p1 = 3.76e-05 . The probability of observing two barcodes within this distance with the same TCR cluster is lower, although this will depend on the size of that particular TCR family. The default can be changed by setting the input parameter –bc *d* where d is the maximum distance within which different barcodes will be collapsed into a single barcode.
